# Enabling Large-Scale Simulations With the GENESIS Neuronal Simulator

**DOI:** 10.3389/fninf.2019.00069

**Published:** 2019-11-15

**Authors:** Joshua C. Crone, Manuel M. Vindiola, Alfred B. Yu, David L. Boothe, David Beeman, Kelvin S. Oie, Piotr J. Franaszczuk

**Affiliations:** ^1^Computational and Information Sciences Directorate, Army Research Laboratory, Aberdeen Proving Ground, MD, United States; ^2^Human Research and Engineering Directorate, Army Research Laboratory, Aberdeen Proving Ground, MD, United States; ^3^Department of Electrical, Computer, and Energy Engineering, University of Colorado, Boulder, CO, United States; ^4^Department of Neurology, Johns Hopkins University School of Medicine, Baltimore, MD, United States

**Keywords:** large-scale simulation, spiking neuronal network, computational neuroscience, multi-compartment neuron model, high performance computing, multiscale modeling

## Abstract

In this paper, we evaluate the computational performance of the GEneral NEural SImulation System (GENESIS) for large scale simulations of neural networks. While many benchmark studies have been performed for large scale simulations with leaky integrate-and-fire neurons or neuronal models with only a few compartments, this work focuses on higher fidelity neuronal models represented by 50–74 compartments per neuron. After making some modifications to the source code for GENESIS and its parallel implementation, PGENESIS, particularly to improve memory usage, we find that PGENESIS is able to efficiently scale on supercomputing resources to network sizes as large as 9 × 10^6^ neurons with 18 × 10^9^ synapses and 2.2 × 10^6^ neurons with 45 × 10^9^ synapses. The modifications to GENESIS that enabled these large scale simulations have been incorporated into the May 2019 Official Release of PGENESIS 2.4 available for download from the GENESIS web site (genesis-sim.org).

## Introduction

Computational models of neurons and neural networks have become an increasingly critical tool in neuroscience. Not only are they used as complementary tools to test theoretical hypotheses and cross-validate experimental data, but they are often a primary mechanism for scientific discovery. Modeling and simulation can be used to probe the fundamental mechanisms of neural networks at spatial and temporal scales well-below current experimental capabilities. Moreover, given the scale and complexity of brain-like neural networks, analytically tractable representations of the network dynamics and functionality are unlikely, further motivating the need for simulation in computational models.

As computational models have become more integrated into the study of the dynamics and function of neural networks, the demand for larger and higher fidelity models has grown. Large scale models containing millions to billions of neurons are necessary to capture the coordinated interaction of multiple brain regions and to represent the respective densities of local and long-range synaptic connections between neurons that are typically found in biological brains. High fidelity neuronal models with complex morphologies are required to accurately represent anatomical and functional variability in neuronal populations and also to portray interactions between local neuronal properties and neural activity at the network level. Understanding the role of non-linear neuronal properties such as intrinsic oscillations, resonance, bursting, and rebounds requires realistic ionic-channel based neuronal models. Likewise, studies of dendritic computation need morphologically detailed neuron models. It is increasingly important to model field potentials that are generated by cortical activity, as measured by scalp or cortical surface electrodes. For this, one needs multi-compartmental neuron models with enough realism in the dendritic morphology and the location of synapses to properly account for the location of the major sinks and sources of currents in the extracellular medium (Kudela et al., 2018).

The multiscale nature of large-scale, high-fidelity neuron modeling, where spatial and temporal scale can vary by many orders of magnitude, introduces a number of computational challenges. These challenges have limited the simulation size, fidelity, and time duration that is computationally tractable. Computational models of single neurons can include hundreds to thousands of coupled ordinary differential equations (ODEs) to account for the complexity of their biology and behavior (De Schutter and Bower, 1994). When combined to form large-scale networks with millions of neurons, the number of ODEs that must be simultaneously solved becomes daunting. Parallel neuronal simulators have been developed (Hines and Carnevale, 1997; Bower and Beeman, 1998; Gewaltig and Diesmann, 2007) that partition the neural network across multiple processing elements (PEs) to speed up computation. However, the discrete nature of neuronal spiking and the complex connectivity of neural networks presents a unique challenge in achieving efficient parallel scalability. In many non-neuronal systems, spatial cutoffs or compact support can be enforced to limit the required communication to PEs representing adjacent spatial regions of the system. In many other systems where long-range interactions are important, methods such as multipole expansion can be employed to reduce both computation and communication costs by exploiting the spatially decaying strength of the long-range interactions (Carrier et al., 1988). However, such approximations are not consistent with what we know about the biology of neural systems. While on average, neurons make on the order of 10,000 synaptic connections to other neurons, the number any given neuron makes can vary from hundreds to hundreds of thousands and are spread over a wide range of spatial scales. This implies that large models of neural networks may require communication between many, if not all, PEs. Furthermore, the interaction between presynaptic and post-synaptic neurons is discrete, as the interaction effects are “on” within some window of the spiking of the presynaptic neuron and “off” otherwise. Because of this discretization, the long-range interactions are not spatially decaying and cannot be homogenized.

Another challenge to large-scale neural models with biologically realistic neuronal models is the wide range of timescales that are relevant to neural behavior, with processes spanning from microseconds (μs) to hours or longer. Simulation of very long timescale behaviors is generally limited by current computational resources, but realistic models of brain behavior at the timescales of organismic behavior—seconds to minutes—require reproducing or otherwise accounting for the dynamics of processes of molecular, cellular, and network processes that operate on shorter timescales. While advanced time integration methods have made significant progress in increasing the required time step size without losing simulation fidelity, capturing the dynamics across this range of timescales can require hundreds of thousands to millions of computational steps, and it is critical that each step is evaluated as efficiently as possible.

To address the computational challenges of modeling neural networks, numerous simulators have been developed over the past two decades. Two of the most actively developed neuronal simulators, NEURON (Hines and Carnevale, 1997) (www.neuron.yale.edu/neuron/), and NEST (Gewaltig and Diesmann, 2007) (www.nest-simulator.org), have optimized for different tradeoffs between fidelity and scale. NEURON is efficient in modeling high-fidelity, biologically realistic neurons consisting of thousands of compartments and has been used extensively in many of the biggest research initiatives on detailed modeling of the brain (Markram et al., 2015; Arkhipov et al., 2018). Recently, NEURON has demonstrated the ability to model ~8 × 10^5^ neurons, represented by an average of 700 compartments per neuron (Kumbhar et al., 2019). In this work NEURON demonstrated excellent scalability on over 30,000 PEs. However, it has not yet been demonstrated that NEURON can scale up to modeling millions of neurons. NEST, on the other hand, has demonstrated the ability to model hundreds of millions of neurons with trillions of synapses while scaling to 10,000's of PEs (Jordan et al., 2018). However, NEST is unable to simulate neurons with complex morphologies, limiting the biological realism of its models. Both NEST and NEURON were recently used for a large scale parallel model of cerebellar cortex with 96,734 cells of several types (Casali et al., 2019). The model was run on parallel computers using the pyNEST and pyNEURON simulators (Eppler et al., 2009; Hines et al., 2009). Both simulators gave similar results and produced firing rates before, during, and after stimulation that were nearly identical. However, the neurons were represented as single-point leaky integrate-and-fire models.

The neuronal simulator, PGENESIS (Bower and Beeman, 1998) (genesis-sim.org), shares many of the benefits of both NEURON and NEST. PGENESIS is a parallel implementation of the GEneral NEural SImulation System (GENESIS). GENESIS and NEURON use the same algorithms and numerical methods for simulations. They have similar modeling capabilities and performance on large, single-cell models (Bhalla et al., 1992; Gleeson et al., 2010). However, we are not aware of any benchmark comparisons for a large network model with multi-compartmental neurons. In spite of these similarities, from the outset, there were major differences in design that influenced the way that the two simulators are used and the way that simulations are created. From the beginning, GENESIS was designed to be object-oriented, with precompiled simulation objects that are linked in a high-level simulation language. This also provides powerful scripting commands for creating and connecting large networks of neurons. This design allows modelers to easily extend the capabilities of the simulator, and to exchange, modify, and reuse models or model components. These objects are organized into libraries, with documentation provided for adding to these libraries or creating new ones. User extensibility is provided at the basic level of writing functions in C to create new objects or commands. This modularity has also made it possible for users with a little programming expertise to easily add new commands or objects to GENESIS. For this reason, GENESIS development has always relied heavily on contributions from users. The GENESIS script interpreter can interact with a running simulation, pausing to make changes, including addition or deletion of simulation elements or messages. This allows interactive development of simulations without quitting to recompile. GENESIS provides a variety of graphical visualization objects that can be linked to other objects in simulation scripts for a run-time display of results. However, most large PGENESIS simulations are run in batch mode with simulation output directed to files. PGENESIS provides parallel versions of objects that can output any simulation variable to files at clocked intervals. These are then analyzed or displayed with external tools such as Matlab or the collection of Python network analysis tools that are provided with GENESIS. Although it would not be difficult to do, we are not aware of any efforts to develop a Python interface for the GENESIS script interpreter. In NEURON, the scripts are written in modified versions of the languages HOC and MOD (NMODL), which are then compiled into C for execution. Model parameters can be changed at run-time, but not the model itself. New channels and other simulation objects can be specified and compiled into a NEURON simulation using NMODL. Extensions to NEURON itself are added by the development team. The NEURON simulation code is not object-oriented, although NEURON now has a Python interface (Hines et al., 2009) that uses an object-oriented model in building simulations, but not at the implementation level.

Another actively developed neuronal simulator for modeling multicompartmental neurons is the Multiscale Object-Oriented Simulation Environment (MOOSE) (https://moose.ncbs.res.in). Currently, most applications are for modeling biochemical reactions and single cell models, for which parallel support is being developed. MOOSE has a robust Python scripting interface, providing a Python-based interface for creating models. It also inherits the GENESIS script parser, giving it backwards compatibility with GENESIS for those objects and commands that have been implemented in MOOSE. As parallel support for network modeling in MOOSE improves, it may also provide a means for providing Python scripting in parallel PGENESIS simulations.

The modularity and multi-compartment solver efficiency of GENESIS, coupled with the sophisticated algorithms of PGENESIS for communicating spikes across PEs, makes PGENESIS an appealing option for performing large-scale, high-fidelity neural simulation. Therefore, it is the goal of this work to assess the viability of PGENESIS for simulating large-scale networks of high fidelity neurons on supercomputing resources.

In this report, we use a high fidelity neural network model that includes structural characteristics of the thalamocortical network (Traub et al., 2005; Kudela and Anderson, 2015; Boothe et al., 2017) to benchmark the performance and scalability of PGENESIS. We identify a number of bottlenecks limiting scalability to large scale networks and present solutions to these bottlenecks. Finally, we evaluate the parallel performance of our modified PGENESIS framework while assessing the effects of various simulation parameters that influence parallel scalability. We demonstrate that PGENESIS is able to efficiently simulate networks with millions of high fidelity model neurons with thousands of connections per neuron.

## Methodology

### Benchmark Model

The benchmark model used in this work was a previously published model of the thalamocortical network (Traub et al., 2005; Kudela and Anderson, 2015; Boothe et al., 2017). Source code for the model used in the present study is available on ModelDB (McDougal et al., 2017) at http://modeldb.yale.edu/260267. The model contained 12 cell types (6 excitatory and 6 inhibitory) organized into layers that correspond to cortical layers two through six in the neocortex of the human brain (Traub et al., 2005). Each cell was represented by 50–74 compartments with each compartment containing up to 14 voltage gated channels. Groups of neurons were organized into microcolumns containing 61 neurons with multiple instances of some neurons. This microcolumnar unit was repeated in the XY plane to generate larger networks. Synaptic connections between neurons were determined by anatomically informed probabilities. Connections between each compartment, neuron type, and cortical layer were assigned a connection probability based on published parameters (Traub et al., 2005). Gap junctions were not included in our model, in contrast with the previously published model, which included both synaptic connections and gap junctions. Since we were simulating a contiguous section of cortical gray matter, distances between neurons were short and probabilities did not vary with distance. This network connectivity scheme is believed to be a worst-case scenario from a parallel communication standpoint, as neurons on each PE were equally likely to be connected to neurons on every other PE. To ensure that the number of synaptic connections per neuron was constant as model size increased, connection probabilities were scaled equally by a factor dependent on the total number of neurons in the simulation.

Maintaining a constant number of connections per neuron while increasing the total number of neurons leads to overall sparser connectivity. Sparser connectivity may lead to a decrease in the average spiking rate. Since communication between neurons in neural simulators is driven by spiking, we also aimed to fix the average spiking rate by driving neurons with an independent Poisson distributed spike train with an average rate of 10 Hz and setting the synaptic weights between neurons to a very low value (1 × 10^−9^ arb. unit). This maintained the computation and communication costs associated with the spiking neurons and ensured that the spiking rate was independent of the network sparsity.

### Simulation Procedures

Simulations were performed with the 2014 Preliminary Release of PGENESIS 2.4 (referred to throughout this paper as “PGENESIS 2.4−2014”) and with our modified version of PGENESIS 2.4 which has been incorporated into the May 2019 Official Release of PGENESIS 2.4 (referred to throughout this paper as “PGENESIS 2.4−2019”). Our modifications were made to increase the number of neurons that can be simulated using PGENESIS and are discussed in detail in section Modifications to PGENESIS. All simulations were performed on Thunder, an SGI ICE X located at the Air Force Research Laboratory (AFRL) DoD Supercomputing Resource Center (DSRC). This machine contains 3,216 compute nodes connected through 4x FDR InfiniBand interconnects with the Enhanced LX Hypercube topology. Each node contains 36 2.3 GHz Intel E5-2699v3 cores that share 126 GB of accessible memory. Both versions of PGENESIS were compiled with the SGI Message Passing Toolkit (MPT) version 2.14 to communicate between PEs.

Partitioning of the neural network across CPU cores was performed by dividing up the microcolumns. Simulations were performed with 1, 4, and 16 microcolumns per core, corresponding to 61, 244, and 976 neurons per core, respectively. To assess the effect of connectivity on computational cost and parallel scalability, simulations were run with 2,000 and 20,000 synaptic connections per neuron. To assess the effect of spiking rate on the computational cost and parallel scalability, simulations were run with independent Poisson distributed spike trains with average rates of 10 and 100 Hz. Since each input spike can lead to 0, 1, or multiple spikes, the actual average spiking rates for all neurons in the simulated network can vary significantly from the input spike train frequencies. Across the network sizes modeled in this work, the average spiking rate ranged from 11.6 to 11.77 Hz for the 10 Hz Poisson input and 46.8–47.5 Hz for the 100 Hz Poisson input. Time integration was performed using the implicit Crank-Nicholson method, employing the Hines (hsolve) method (Hines, 1984). A time step size of 25 μs was used for all simulations.

Two measures were used for evaluating the performance and scalability of PGENESIS: wall clock time (*T*) and weak scaling efficiency (*E*). For weak scaling studies, the problem size per CPU core was fixed and the number of cores was increased from a minimum number, *m*. Weak scaling efficiency on *n* CPU cores was then defined as

(1) $$\begin{array}{l} E\left(n\right)=\;\frac{T_{m}}{T_{n}}\;\forall \;n\geq m \end{array}$$

where *T*_*n*_ is the wall clock time on *n* CPU cores and *T*_*m*_ is the wall clock time on *m* CPU cores. In this work, *m* was chosen such that the number of neurons in the system was on the order of the number of connections per neuron. This was to prevent simulations where each neuron was connected to every other neuron numerous times, which is unrealistic. A weak scaling efficiency of 1 corresponds to ideal performance and lower values of *E* correspond to lower parallel efficiency.

### Modifications to PGENESIS

Early in our evaluation of the scalability of PGENESIS, we encountered three issues which inhibited our ability to complete the assessment. In this section, we describe the issues, as well as our modifications made to PGENESIS to address these issues. These improvements have been merged into the May 2019 Official Release (PGENESIS 2.4−2019). The combined GENESIS/PGENESIS 2.4 Official Release distribution may be downloaded from the GENESIS web site (http://genesis-sim.org).

### Repeatable Connectivity

**Figure 1:**
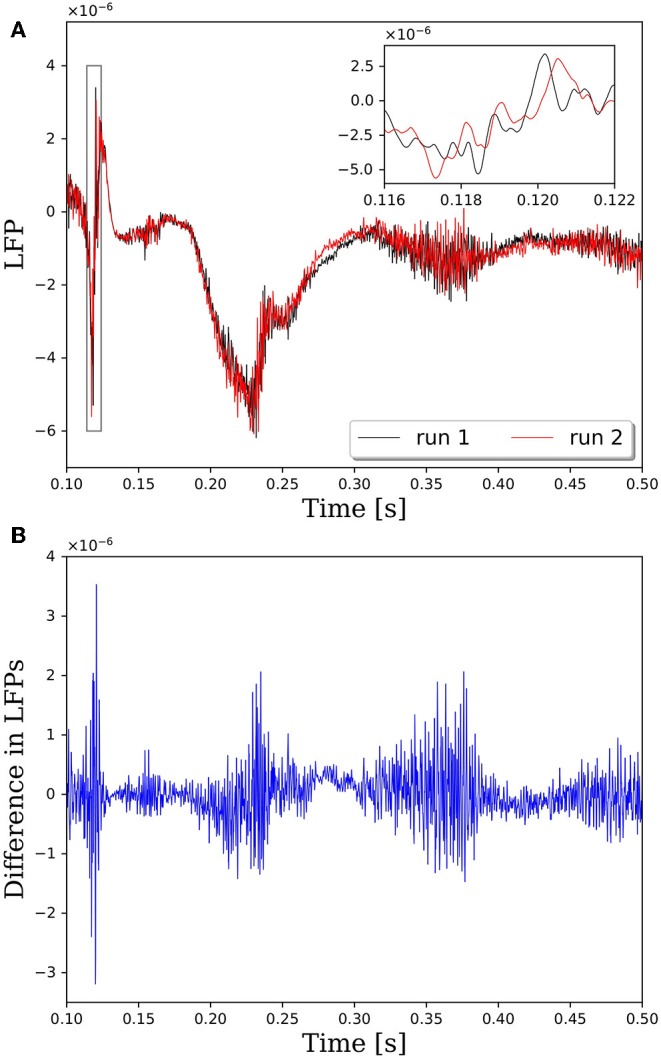
Comparison of local field potentials (LFPs) between two simulations with identical inputs on PGENESIS 2.4 – 2014. Model consists of 15,616 neurons partitioned on 256 CPU cores with a connection density of 2,000 synapses per neuron. **(A)** Raw LFP output measured ~6 μm above cortical patch. The plot insert corresponds to a magnified view of the window indicated by the gray box. **(B)** Difference in LFPs between simulations.

Repeatability is a critical property of computational algorithms and tools. Without repeatability it is difficult, if not impossible, to assess if changes to the system response are due to intentional changes in the model or due to the inherent variability in the assignment of synaptic connections. In PGENESIS 2.4−2014, the standard way in which connections are made between neurons on remote cores does not lead to repeatable network connectivity. To demonstrate the effect of variable connectivity on the simulation output, we performed repeated simulations with identical input commands to generate the neural network model and identical random seeds for the Poisson distributed spike train input. Note that in these simulations we set the synaptic weights to 1, rather than 1 × 10^−9^ as done in all other simulations in this report. This caused the network to be driven more by activity in the neurons as opposed to the Poisson distributed spike train input. The variability is illustrated in Figure 1, where the local field potentials (LFPs) from two simulations with identical input are plotted in Figure 1A. Taking the difference between LFPs in Figure 1B reveals that the variability is on the same order as the LFP. And while these LFPs look qualitatively similar in this case, the magnified view from the insert in Figure 1A suggests that the spike timing between runs may vary by up to 1 ms. It has been shown that small perturbations in the spike timings of individual neurons can be amplified to large deviations in the macroscopic behavior due to the complex, non-linear response of neural networks (London et al., 2010).

**Figure 2:**
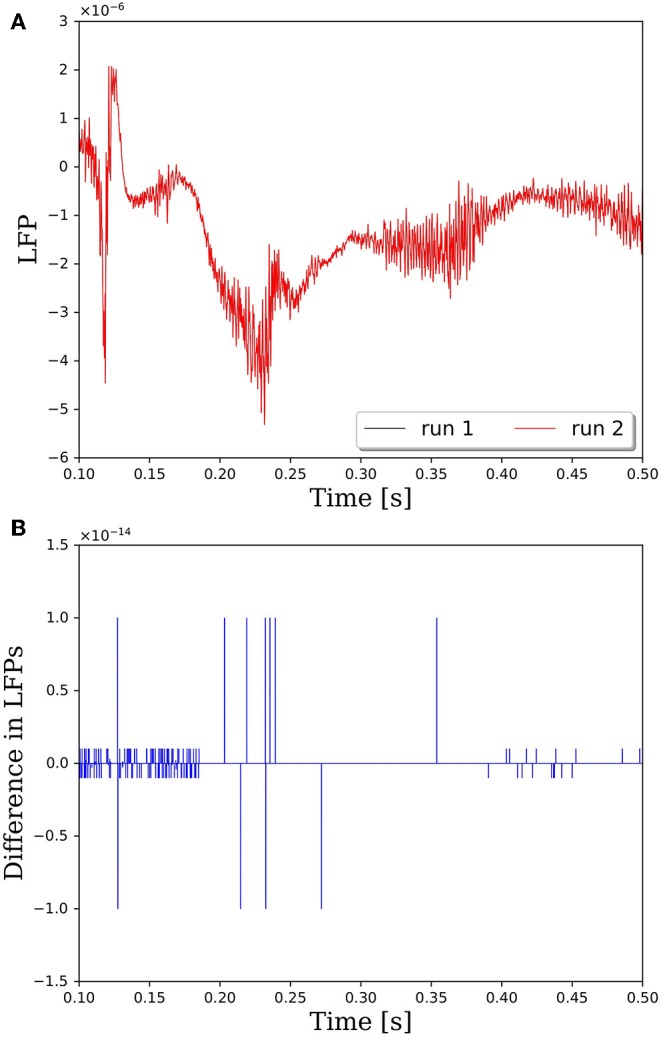
Comparison of LFPs between two simulations with identical inputs after modifying the *rvolumeconnect()* function. Model consists of 15,616 neurons partitioned on 256 CPU cores with a connection density of 2,000 synapses per neuron. **(A)** Raw LFP output measured ~6 μm above cortical patch. **(B)** Difference in LFPs between simulations.

The reason that network connectivity is not repeatable for parallel simulations in PGENESIS is due to the way random connections are made within the *rvolumeconnect()* function. When a connection probability is provided to *rvolumeconnect()*, the core containing the source neuron sends a message to the core containing the destination neuron. The destination core then generates a random number and compares it to the connection probability to determine if the connection should be made. This leads to a race condition where even if *rvolumeconnect()* is called in the same order every time, the destination cores will process each potential connection pair in the order received, which is not guaranteed to be consistent from one simulation to the next. This will lead to different random numbers being generated for each pair and, therefore, different connectivity. To address this issue, we have changed *rvolumeconnect()* to generate the random number on the source core, which ensures that as long as *rvolumeconnect()* is called in the same order, the resulting network connectivity will be identical. Figure 2 shows that after modifying *rvolumeconnect()*, variations in LFPs are on the order of machine precision. We note that repeatability is only guaranteed for a fixed number of CPU cores and fixed partitioning of the neural network.

### Memory Leaks

**Figure 3:**
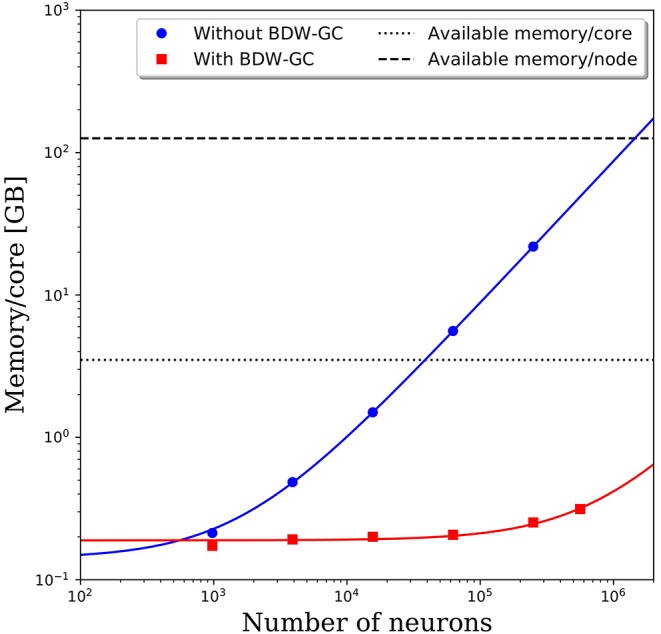
Memory usage per CPU core with and without the BDW-GC. The symbols represents measurements from simulations. The solid lines represent linear fits to the data. Dashed and dotted lines are the available memory on the Thunder system.

When attempting to scale PGENESIS 2.4−2014 to large networks, memory quickly became the limiting factor. This is demonstrated by the blue circles in Figure 3, which show the memory usage per core with increasing network size while maintaining 61 neurons per core and 2,000 connections per neuron. Despite a fixed number of neurons and connections per core, we observed a substantial increase in the memory usage per core. This became a significant hindrance to scalability when the memory usage per core surpassed the available memory per core, which is 3.5 GB on Thunder and is indicated by the dotted line in Figure 3. The crossover point for this benchmark model was only ~40,000 neurons. Beyond this network size, we were forced to reduce the number of cores per node so that each core could use a larger portion of the 126 GB of shared memory. This significantly increased the computational cost. For example, the 250,000 neuron simulation in Figure 3 was partitioned onto 4,096 cores, but had a memory requirement of almost 22 GB per core, limiting the number of active cores per node to 5. With a limit of 5 cores per node, the 4,096 cores had to be divided amongst 820 nodes, effectively requiring 29,520 cores. This discrepancy would only grow as the neural network increased in size, requiring more memory per core. A linear least-squares fit to the data is indicated by the solid blue line in Figure 3. Assuming that the linear growth in memory usage continues beyond 250,000 neurons, it is projected that by 1.4 million neurons, the memory usage per core would be too big to run, even if limited to a single core per node. Due to the rapidly growing memory usage, PGENESIS 2.4−2014 cannot efficiently run on high performance computing resources (HPC) at the DSRC or many other supercomputing resource centers.

Memory profiling of PGENESIS 2.4−2014 suggested that most of the increased memory usage was due to memory leaks, rather than necessary increases in storage. An initial attempt to fix the various memory leaks proved to be tremendously labor intensive. To address the memory leaks more efficiently, we integrated the Boehm-Demers-Weiser garbage collector (BDW-GC) (www.hboehm.info/gc/) into PGENESIS. The BDW-GC replaces standard memory allocation calls and automatically recycles memory when found to be inaccessible. The red squares in Figure 3 illustrate the significant reduction in memory cost after integrating the BDW-GC. Without the garbage collector, a simulation of 500,000 neurons was computationally intractable due to the large memory requirement limiting the active CPU cores per node to two. With the BDW-GC, the memory usage is reduced by two orders of magnitude, enabling full use of each node. A linear least-squares fit to the data is indicated by the solid red line in Figure 3. The plot suggests that the memory usage still grows linearly with increasing neural network size, however, the rate of growth has decreased dramatically. Assuming the linear scaling in memory usage with the number of neurons continues, the memory per core limit would not be reached until ~15 million neurons. Furthermore, the limiting system size at which the memory requirements surpass 126 GB would increase to over 550 million neurons.

**Figure 4:**
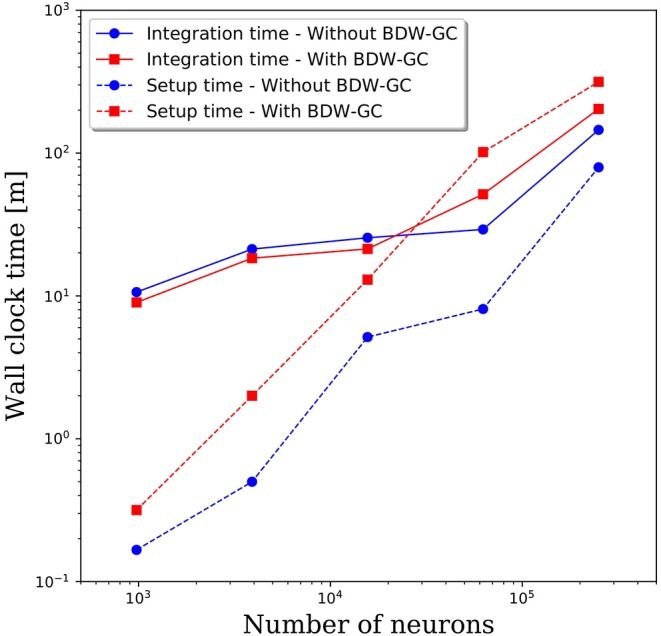
Wall clock time for model setup and to integrate 1 s of simulation time with and without the BDW-GC.

The added overhead with any garbage collector is always a concern. To assess the added cost of the BDW-GC, we compared the wall clock time for setup and to integrate 1 simulated second. The results in Figure 4 reveal that both setup time and simulation time are slower for large networks with the BDW-GC. However, the simulation time is only 40% slower for the 250,000 neuron simulation, and is faster for network sizes up to 15,000 neurons. The wall clock time for setup of the 250,000 neuron simulation increased by a factor of 4 with the BDW-GC. Whether this is an acceptable increase in time will depend on the user and simulation parameters. For example, if simulating large networks for fractions of a second, the setup time will dominate and the cost of the BDW-GC will be significant. However, this effect will be much smaller if simulating a large network for tens of seconds. We view the added cost of the BDW-GC to be an acceptable tradeoff given the savings achieved by utilizing all of the cores on the node and by enabling simulations that could not otherwise be performed, even if utilizing a single core per node. However, the BDW-GC is easily deactivated at compile time in PGENESIS 2.4−2019, simply by omitting the GCMALLOC flags in the well-documented Makefile. This gives the user the ability to decide if the benefits of the BDW-GC outweigh the costs. We also emphasize that the benefits of the BDW-GC demonstrated in Figure 3 could be achieved without the added costs demonstrated in Figure 4 by individually correcting the memory leaks in GENESIS and PGENESIS, though it appears that this would be a labor intensive process.

### Integer Overflows

Another issue encountered when trying to scale the PGENESIS 2.4−2014 simulator to larger network sizes was an integer overflow error, which occurred when the number of remote messages became too large. Within the simulation parameters used in this work, the integer overflow error occurred when trying to model 244 neurons per core with 20,000 synaptic connections per neuron, resulting in ~4.9 million synaptic connections per core. A systematic study would be required to determine the exact parameter space in which this error occurs. However, we did not observe this error when simulating up to 1.2 million connections per core. The integer overflow is located in the buffer manager code, which contains variables of type short that are responsible for tracking remote messages. By changing the variables types to 32-bit integers, the overflow issue was eliminated for all simulation parameters used in this work.

## Performance and Scalability Results

Using our modified version of PGENESIS, we investigated the parallel performance for the benchmark thalamocortical model. The baseline model parameters were 2,000 synaptic connections per neuron and 10 Hz Poisson distributed spike train input. The performance of this baseline model is presented in section Partitioning Across CPU Cores. In the subsequent sections, we varied the connection density and spiking rate to explore the effects of these parameter changes on the performance of the modified version of PGENESIS.

### Partitioning Across CPU Cores

**Figure 5:**
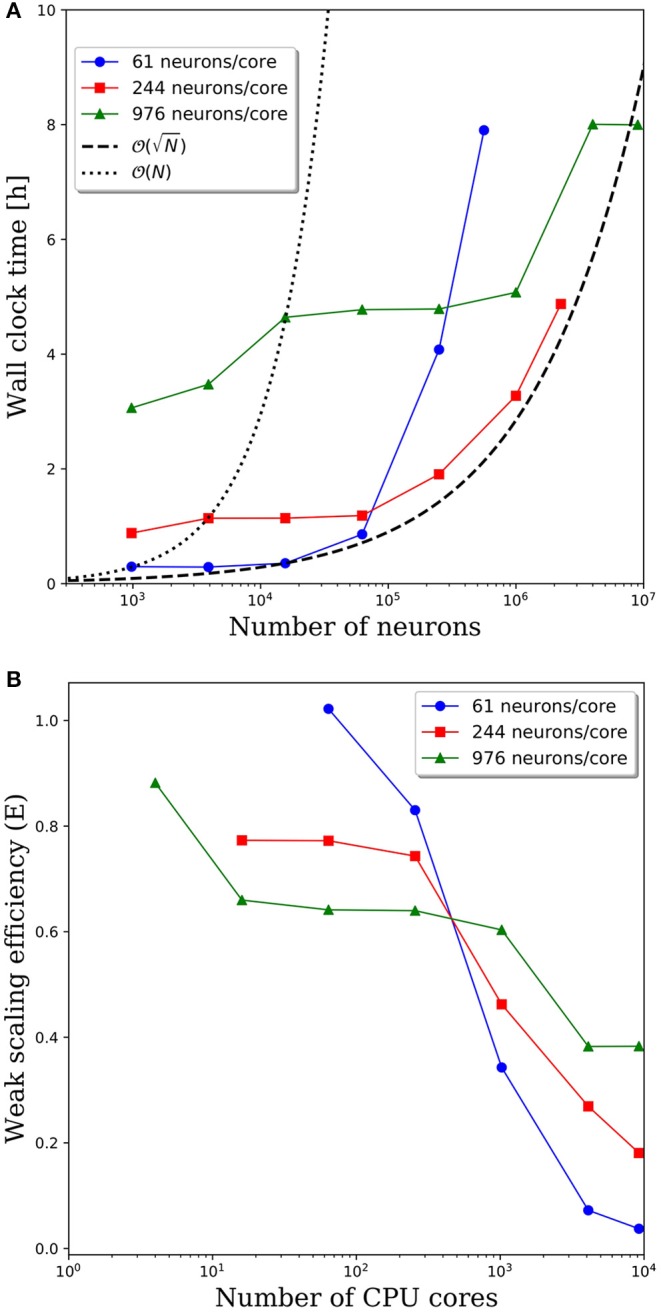
**(A)** Wall clock time for simulations of neural networks with 2,000 synapses per neuron. **(B)** Weak scaling efficiency, defined in Equation 1, for the timings in **(A)**.

Wall clock times to integrate 1 simulation second, and corresponding weak scaling efficiencies, are presented in Figures 5A,B, respectively, for multiple network decompositions with varying number of neurons per core. For each decomposition, the minimum network size is 976 neurons which corresponds to a minimum number of CPU cores (*m*) of 64, 16, and 1 for 61, 244, and 976 neurons/core, respectively. For small network sizes up to ~50,000 neurons, we observe that partitioning the network onto more cores is advantageous, as the wall clock time is significantly smaller for the 61 neurons/core partitioning. At ~50,000 neurons, the communications cost begins to dominate and the wall clock time rises rapidly for the 61 neurons/core partitioning. For each partitioning, the weak scaling efficiency drops significantly between 256 and 1,024 cores. However, even in this regime there are clear benefits to further parallelization. Take, for example, the 244 neurons per core partitioning; when scaling from ~250,000 neurons (1,024 cores) to ~2.25 million neurons (9,216 cores), the wall clock time only increased by a factor of 2.5, despite a 9x increase in the system size.

For this particular model and range of network sizes, we observe an approximate $\text{O}(\sqrt{N})$ scaling of the minimum wall time with respect to the partitioning of neurons per core, where N is the total number of neurons in the system. This scaling is illustrated by the black dashed line in Figure 5A. While constant wall clock time would indicate ideal scalability, this is unrealistic for large neural networks given the non-local nature of synaptic connections. Yet, the benefits of parallelization are clear when one considers the best possible scaling when increasing the system size on a fixed number of core is O(N), indicated by the black dotted line in Figure 5A. At that scaling rate, simulations of millions to tens of millions of neurons would be computationally intractable.

### Synaptic Connection Density

**Figure 6:**
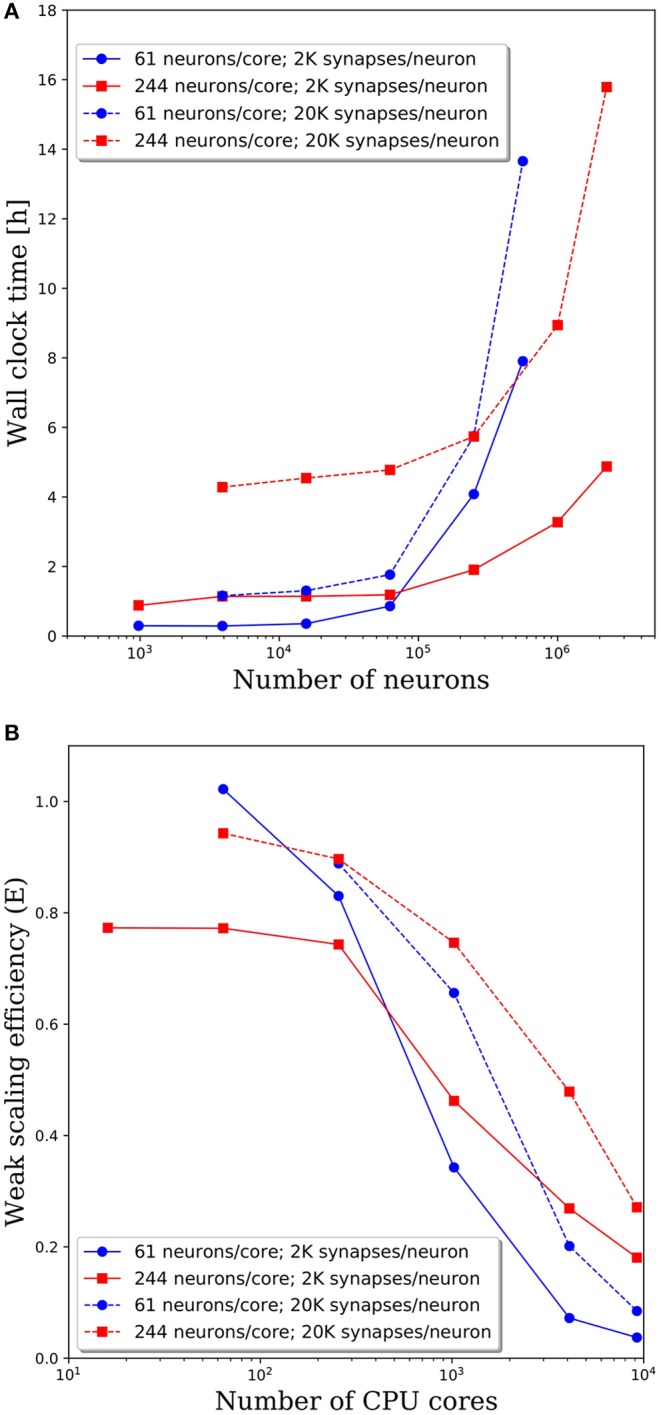
**(A)** Wall clock time for simulations of neural networks with 2,000 and 20,000 synapses per neuron. **(B)** Weak scaling efficiency, defined in Equation (1), for the timings in **(A)**.

To investigate the effect of connection density on the time to simulate 1 s of neural activity, we increased the number of synaptic connections per neuron from 2,000 to 20,000. We emphasize that, despite the higher connection density, the spiking rate remained fixed due to the negligible synaptic weights as described in section Methodology. Due to the higher connection density, the minimum network size was increased to 3,904 neurons. A comparison of wall clock times is shown in Figure 6A and reveals that simulation times increased significantly with a higher connection density. For partitions of 61 and 244 neurons/core, the wall clock time increased by a factor of 2–4 for a 10x increase in the number of synaptic connections. This increase is not surprising given the added cost associated with routing each spike to more local and remote neurons at the higher connection density.

The weak scaling efficiencies observed in Figure 6B show an improvement in weak scaling efficiency with increasing connectivity. It is reasonable to expect that a higher connection density would lead to higher communication cost, reducing parallel efficiency. However, even at 2,000 connections per neuron, it is likely that neurons will need to communicate with neurons on most remote CPU cores. Whether that spike must be communicated to one neuron or 10 neurons on the remote core has limited effects on the communication costs. It is likely that in the dilute limit of connection density, a notable effect on the parallel efficiency would be observed. Yet in the biologically realistic regime of thousands to tens of thousands of synaptic connections per neuron, we observe a significant effect on the wall clock time, but not on parallel efficiency.

### Spiking Rate

**Figure 7:**
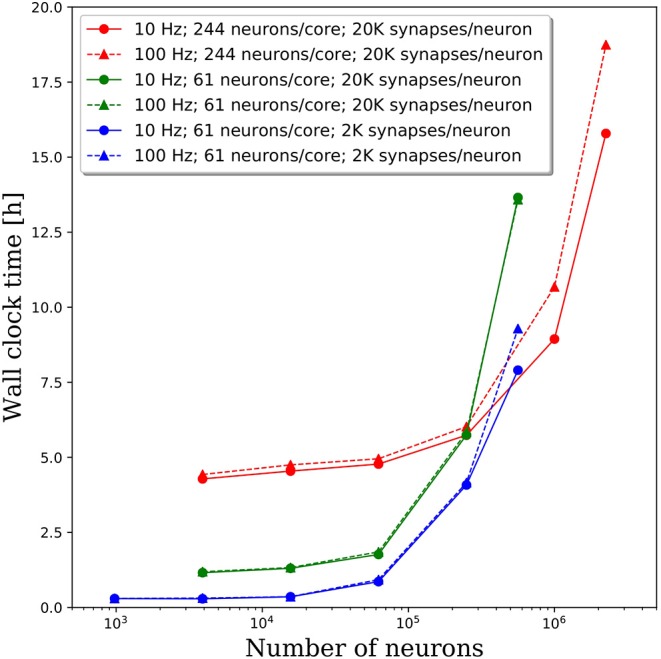
Effect of spiking rate on the wall clock time to integrate 1 simulation second. The solid lines correspond to the original 10 Hz Poisson distributed spike train inputs which cause an average spiking rate of 11.7 Hz. The dashed lines correspond to 100 Hz Poisson distributed spike train inputs which cause an average spiking rate of 47 Hz.

We increased the spiking rate from 11.7 to 47 Hz by increasing the average frequency of the Poisson distributed spike train input to each neuron from 10 to 100 Hz. The resulting wall clock times to integrate 1 simulated second are shown in Figure 7. We find that for network sizes up to ~500,000 neurons, the effect of spiking rate is negligible. Even for network sizes beyond 500,000 neurons, the wall clock time increased by <20% despite a 4x increase in spiking rate. These results are surprising as we expected that both the frequency of communication and the amount of communicated data would increase, resulting in longer walk clock times for simulations. A more thorough investigation of the communication algorithms employed in PGENESIS and networking configuration of the messaging passing interface (MPI) implementation are required to explain why the wall clock time is sensitive to connection density yet relatively insensitive to spiking rate.

### Setup Time

**Figure 8:**
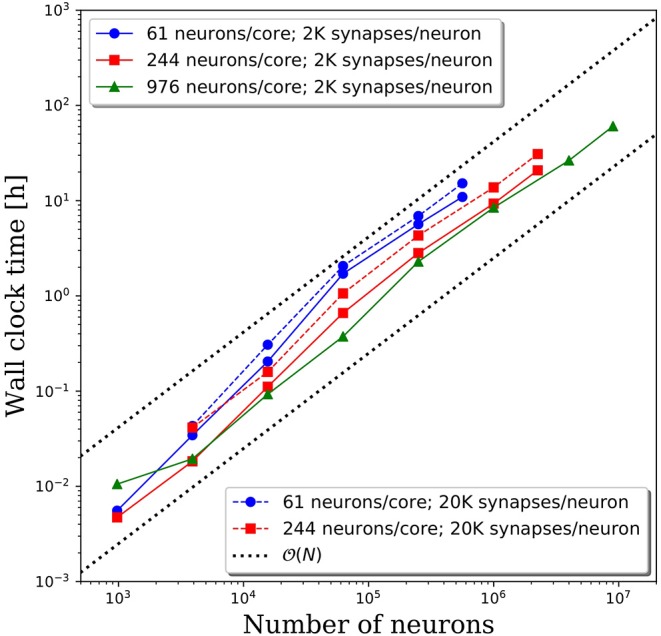
Wall clock time to perform model setup.

As illustrated in Figure 4, the setup time was insignificant for small neural networks, but can dominate the computational cost when scaling to large networks. The majority of this cost was in establishing 10^8^-10^9^ synaptic connections between neurons. In Figure 8, we plot the setup times for all network sizes, decompositions, and connection densities presented in this report. For all simulation parameters, the setup times scaled as approximately O(N) with the number of neurons. As expected, fewer neurons per core and higher connection densities led to longer setup times, because more remote connections must be established. For network sizes up to 100,000 neurons, the setup time was on the order of 1 h or less, which should be negligible for most simulation durations. However, when the network size surpassed one million neurons, the setup time became significant. As discussed in section Memory Leaks, removing the garbage collector by individually eliminating the memory leaks may significantly reduce the setup time. However, it is unlikely to reduce the O(N) scaling. To improve the scaling, more sophisticated algorithms are required for initializing billions of synaptic connections across neurons on all cores.

## Discussion

The use of multi-compartment neuronal models increases the computational requirements per neuron, but provides significantly higher biological fidelity than the integrate-and-fire or single-compartment Hodgkin-Huxley neurons that have been used in most other large-scale neural network models (Lytton et al., 2016; Jordan et al., 2018). We are focused on the simulation of large-scale models with higher neuronal complexity for two related reasons. First, there has been an increased acceptance that brain processes are the product of distributed sub-networks (Sporns and Betzel, 2016) within a large network of sparsely-connected, heterogeneous neurons. That significant heterogeneity exists in the brain is self-evident. Neurons vary in the details of their morphology; the number, strength, targets, and spatial extent of their connections; and in the neurochemistry that governs their synaptic interactions, such that no two neurons, or their behavior, are ever likely to be identical. Second, recent years have also witnessed the expanded use of parallel, high-performance computing (HPC) architectures to enable simulation of neural networks at scales comparable to that of the biological brain, including the human brain.

The impact of neuronal heterogeneity remains an open question. Theoretical investigations have demonstrated both increased and decreased synchronization when the heterogeneity of nodes in small network models is varied. For example, Zhang and colleagues examined small systems (*N* < 10) of coupled oscillators with symmetric connectivity. They showed analytically that some systems exist within this class where heterogeneity in system nodes is necessary for stable synchronous dynamics, which cannot be achieved when the nodes are homogeneous (Zhang et al., 2017). Conversely, increasing the heterogeneity of both the intrinsic frequency of neuron-like oscillators (Tsodyks et al., 1993; *N* = 100) and neuronal excitability (i.e., Ca^2+^ conductance) (Golomb and Rinzel, 1993; *N* = 1,000) leads to asynchronous, rather than synchronous, activity in the network. Similar results have been shown with increasing heterogeneity of the number of connections (Golomb and Hansel, 2000; *N* = 800) and connection weights (Denker et al., 2004; *N* = 100). Lengler et al. have also argued that variance in the reliability of synaptic transmission not only diminishes synchronization in recurrently-connected neural network models, but has important, if somewhat counterintuitive, functional advantages, including improved speed, sensitivity, and robustness (Lengler et al., 2013). Together, the potentially large number of parameters across which neurons vary and the decreasing analytic tractability of the brain as the numbers of parameters and neurons increases points toward the importance of large-scale simulation models with increasing neuronal complexity.

High-performance computing resources have enabled many large scale simulation of millions (Eliasmith et al., 2012) to billions (Izhikevich and Edelman, 2008; Jordan et al., 2018) of neurons. However, each neuron in these networks were represented by simple, single compartment models. Among the most extensive assessments of the current capabilities for large scale simulations of high fidelity neuronal models is the recent work of Kumbhar et al. (2019). They optimized the compute engine of NEURON for modern multi-core computing architectures and examined the parallel scalability for models of varying complexity. The largest model was of the rat hippocampus and contained ~8 × 10^5^ neurons, an average of 700 compartments per neuron, and 450 synapses per neuron. It is difficult to make direct comparisons between the simulation timings of that NEURON rat hippocampus model and the GENESIS Traub thalamocortical model used in this work. The number of compartments per neuron in the rat hippocampus model was an order of magnitude larger than the Traub model, while the number of synapses per neuron was an order of magnitude smaller. Therefore, one would expect the computational cost of the rat hippocampus model to be significantly higher, but the communication costs to be significantly lower. Despite these differences, it is still informative to qualitatively compare the simulation timings of the two models. Using 4,096 cores, NEURON was able to simulate 1 biological second in 4.5 h, which is 40% longer than the 3.25 h required by GENESIS to simulate 1 million neurons of the Traub network on the same number of cores. However, Kumbhar et al. showed excellent scalability and were able to reduce that time to under an hour on 32,768 cores. Increasing the number of cores for GENESIS to simulate 1 million neurons beyond 4,096 cores resulted in an increased simulation time, suggesting communication costs were dominating the computation. These results are consistent with what would be expected given the differences in model complexity discussed above. A more direct comparison of simulation timings can be made with the original Traub model (Traub et al., 2005), although our model does differ from the original Traub model, particularly in the absence of gap junctions. Traub found that it took 18.75–21.2 h to simulate 1 s at a time step of 2 μs with a network of 3,560 neurons partitioned across 14 cores. Due to the presence of gap junctions in their model, they required a significantly smaller time step than the 25 μs used in this work. Normalizing their wall clock time by a factor of 12.5 to account for the differences in time step size, we can approximate the equivalent timing of the original work of Traub to 1.5–1.7 h per simulated second. The closest comparison in our work is a simulation of 3,904 neurons partitioned on 16 cores which required 1.14 h per simulated second which is approximately a 30% reduction. The same model partitioned across 64 cores required only 17 min. For a detailed discussion of several other large-scale simulation efforts, see also (De Garis et al., 2010; Fan and Markram, 2019).

Together, the focus on large-scale neural networks with high neuronal complexity and the increasing use and availability of high performance computing resources for simulating such systems points toward a need for usable frameworks to enable modeling efforts. The use of common frameworks across such efforts will be increasingly important, as reproducibility becomes a larger focus (McDougal et al., 2016). We have shown that by modifying PGENESIS, we were able to efficiently simulate networks with millions of high fidelity model neurons with thousands of connections per neuron: with a connection density of 2,000 synapses/neuron, PGENESIS 2.4−2019 is able to integrate 1 simulated second for 100,000 neurons in ~1 h, 1 million neurons in ~3 h, and 9 million neurons in ~8 h. This compares favorably to a runtime estimate reported in Eliasmith et al. (2012) of 2.5 h processing time for 1 s of simulated time.

Finally, several groups are developing neuromorphic capabilities that are enabling models that surpass even the human brain in scale and performance. For example, IBM Research developed a simulator of its TrueNorth neuromorphic processor, Compass, providing initial performance of a model with 0.65 × 10^11^ neurons and 0.16 × 10^14^ connections running on an IBM Blue Gene/Q supercomputer (Preissl et al., 2012). The next year, those numbers had increased to 5.3 × 10^11^–over five times the number of neurons in the human brain—with an almost 10-fold increase to 1.37 × 10^14^ connections (Wong et al., 2012). The European Union's Human Brain Project has developed the BrainScaleS system, a neuromorphic hardware platform that utilizes analog circuits and has achieved execution speeds that are 10,000 times faster than biological brains in models with as many as 4 × 10^6^ neurons and 10^9^ synapses. The emergence of such alternative computing platforms puts an even greater premium on achieving better understandings of the behavior of large-scale neural networks, and makes the availability of computationally efficient frameworks for simulating large-scale networks of complex, heterogeneous elements increasingly urgent.

## Conclusion

In this report, the viability of PGENESIS for performing large-scale simulations of high fidelity neuronal models has been evaluated. We discovered issues with the 2014 version of PGENESIS 2.4, which prevented it from scaling to large network sizes on HPC resources. By modifying PGENESIS to address issues with repeatability, memory usage, and integer overflows, we significantly increased the network size that is computationally tractable with this simulation system.

Using our modified version of PGENESIS and a thalamocortical network model as a benchmark, simulation performance and scalability was evaluated. The benchmark model contained 12 different types of neurons (e.g., pyramidal cells, interneurons, etc.). Different neuron types had between 50 and 74 compartments. We demonstrate that with HPC resources, we can tractably simulate high fidelity neural networks with 9 × 10^6^ neurons at 2,000 connections per neuron (18 × 10^9^ synapses) and 2.2 × 10^6^ neurons at 20,000 connections per neuron (45 × 10^9^ synapses). These modifications are included in the May 2019 Official Release of PGENESIS 2.4, available for download from the GENESIS web site (genesis-sim.org).

## Data Availability Statement

The datasets generated for this study are available on request to the corresponding author. The GENESIS model scripts are also available on ModelDB at: http://modeldb.yale.edu/260267.

## Author Contributions

All authors listed have made a substantial, direct and intellectual contribution to the work, and approved it for publication.

### Conflict of Interest

The authors declare that the research was conducted in the absence of any commercial or financial relationships that could be construed as a potential conflict of interest.
